# The problem of pulmonary arterial hypertension in end-stage renal disease: can peritoneal dialysis be the solution

**DOI:** 10.1186/s12882-022-02998-y

**Published:** 2022-12-05

**Authors:** Abdullah K. Alhwiesh, Ibrahiem Saeed Abdul-Rahman, Abdullah Alshehri, Amani Alhwiesh, Mahmoud Elnokeety, Syed Essam, Mohamad Sakr, Nadia Al-Oudah, Abdulla Abdulrahman, Abdelgalil Moaz Mohammed, Hany Mansour, Tamer El-Salamoni, Nehad Al-Oudah, Lamees Alayoobi, Hend Aljenaidi, Ali Al-Harbi, Dujanah Mousa, Abdulghani Abdulnasir, Sami Skhiri

**Affiliations:** 1 Nephrology Division, Department of Internal Medicine, King Fahd Hospital of the University, Imam Abdulrahman Bin Faisal University, Al-Khobar, 1952 Saudi Arabia; 2 Cardiology Division, Department of Internal Medicine, King Fahd Hospital of the University, Imam Abdulrahman Bin Faisal University, Dammam, Saudi Arabia; 3 Department of Electrical Engineering, Queen’s University, Toronto, Kingston, Canada; 4 Diaverum Al-Majdoie Dialysis Center, Dammam, Eastern Province Saudi Arabia

**Keywords:** Pulmonary arterial hypertension, Automated peritoneal dialysis, ESRD, ECHO cardiography

## Abstract

**Background:**

Pulmonary arterial hypertension (PAH) in the setting of end-stage renal disease (ESRD) has important prognostic and therapeutic consequences. We estimated the prevalence of PAH among patients with ESRD treated with automated peritoneal dialysis (APD), investigated the effect of different variables and compared pulmonary artery pressure and cardiac function at the beginning and end of the study.

**Methods:**

This is a 5-year study in which 31 ESRD patients on APD were recruited after fulfilling inclusion criteria. Blood samples were collected from all patients for the biochemical and hematological data at the beginning of the study and every month and at the study termination. Total body water (TBW) and extracellular water (ECW) were calculated using Watson’s and Bird’s calculation methods. All patients were followed-up at 3-month interval for cardiac evaluation. Logistic regression analysis was used to assess the relation between different variables and PAH.

**Results:**

The mean age of the study population (*n* = 31) was 51.23 ± 15.24 years. PAH was found in 24.2% of the patients. Mean systolic pulmonary artery pressure (sPAP) and mean pulmonary artery pressure (mPAP) were significantly higher in the APD patients at study initiation than at the end of the study (40.75 + 10.61 vs 23.55 + 9.20 and 29.66 + 11.35 vs 18.24 + 6.75 mmHg respectively, *p* = 0.001). The median ejection fraction was significantly lower in patients with PAH at zero point than at study termination [31% (27-34) vs 50% (46-52), *p* = 0.002]. Hypervolemia decreased significantly at the end of study (*p* <  0.001) and correlated positively with the PAP (r = 0.371 and r = 0.369), p = 0.002). sPAP correlated with left ventricular mass index, hemoglobin level, and duration on APD.

**Conclusions:**

Long term APD (> 1 years) seemed to decrease pulmonary arterial pressure, right atrial pressure and improve left ventricular ejection fraction (LVEF). Risk factors for PAH in ESRD were hypervolemia, abnormal ECHO findings and low hemoglobin levels. Clinical and echocardiographic abnormalities and complications are not uncommon among ESRD patients with PAH. Identification of those patients on transthoracic echocardiography may warrant further attention to treatment with APD.

## Background

End-stage renal disease (ESRD) is a worldwide health problem, however, only about 20% of the world’s ESRD patients have access to renal replacement therapies and these therapies are still associated with severely reduced quality of life, high healthcare costs and high rates of sudden-death [1–3]. Hemodialysis (HD) is associated with higher adjusted mortality (12.7%) compared to peritoneal dialysis (PD). Further, the annual payer cost for PD is also lower than HD and PD exhibits survival advantages over HD in short-, medium- and long-term outcomes [4–6]. Treatment choice for ESRD is further complicated by the presence of serious comorbidities. ESRD patients often exhibit high risk for cardiovascular diseases. Cardiovascular complications, including pulmonary arterial hypertension (PAH), are the major cause of mortality in ESRD patients undergoing dialysis [7–9]. PAH is defined as an abnormally high blood pressure in the pulmonary artery, pulmonary vein or pulmonary capillaries, and is a chronic and progressive disease that results in right heart failure and sudden death if left untreated [10]. Importantly, 30–50% of CKD and ESRD patients have PAH and the risk factors for ESRD-associated PAH include altered endothelial function, increased cardiac output (CO), myocardial defects and left heart dysfunction [11]. High prevalence of PAH is observed in ESRD patients undergoing chronic HD or conservative treatment, and PAH in these patients is associated with enlarged left atrium, elevated thromboxane B2 and N-terminal pro-brain natriuretic peptide (NT-proBNP) levels and abnormal left ventricular diastolic diameter [8–13]. Although the precise mechanisms remain unknown, it is proposed that PAH in ESRD is caused by diastolic dysfunction, volume overload, left ventricular disorders, sleep disorder, dialysis membrane exposure, endothelial dysfunction and vascular calcification, the pulmonary vascular stiffness and vasoconstriction that is unable to accommodate to and the increased cardiac output caused by anemia and hypervolemia [13–17].

Nevertheless, few studies investigated the incidence of PAH in ESRD patients undergoing automated peritoneal dialysis (APD) or examined the risk factors promoting PAH in these patients. In addition, results of previous studies were not consistent, and studies were mostly retrospective. Considering the lack of information regarding PAH in chronic APD patients, we investigated the prevalence of PAH in ESRD patients undergoing APD in our center by collecting detailed information on general data, biochemical parameters and echocardiographic findings. Further, risk factors for PAH were assessed from the collected data to provide the theoretical basis for future studies.

## Methods

Between February 2015 and March 2020, 128 ESRD patients were treated with APD therapy at the Dialysis Center of King Fahd Hospital of the University, Saudi Arabia. The 128 patients included 85 males (66.4%) and 43 (33.6%) females (mean age, 54.94 ± 14.42 years; age range, 18–75 years; mean dialysis time, 36.22 ± 13.52 months). After obtaining study-related approvals from the Ethics committee of King Fahd Hospital, written informed consent to participate in and to publish the study was also obtained from all patients or their legal guardians. Study protocols conformed to the ethical principles of medical research involving human subjects based on the Helsinki Declaration.

**Fig. 1:**
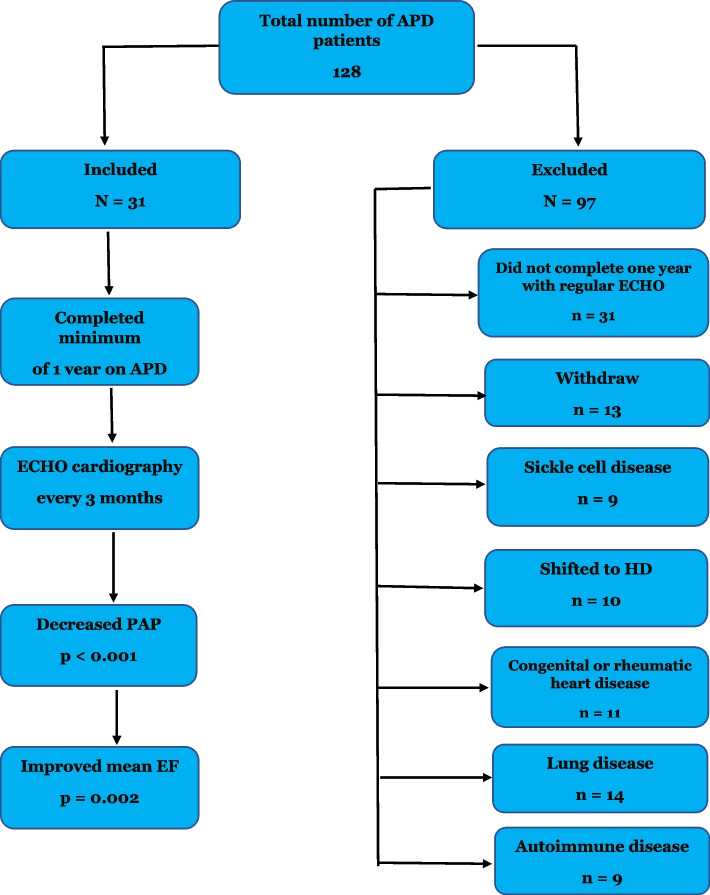
Flow diagram for patients’ population: APD: automated peritoneal dialysis, ECHO: ECHO cardiography, PAP: pulmonary artery pressure, EF: ejection fraction, HD: hemodialysis, Lung disease: chest wall, parenchymatous lung disease and pulmonary embolism

Study inclusion criteria (Fig. 1) were (1) patients undergoing APD with a daily cumulative dialysate dose of 10–15 L, (2) age > 18 years, (3) patients receiving renal replacement therapy (APD) for more than 12 months with stable disease, and (4) patients with complete clinical data on laboratory tests and echocardiography results. Exclusion criteria (Fig. 1) were (1) patients with congenital heart diseases, rheumatic heart disease, valvular heart disease, HIV, chronic obstructive pulmonary disease, chest wall or lung parenchymal disease and pulmonary embolism or autoimmune diseases (systemic lupus erythematosus, rheumatoid arthritis, scleroderma and polyangiitis) and (2) patients who previously received HD). (3) In addition, we considered important to exclude patients with sickle cell disease since this disease has a relatively high prevalence of PAH. Patients who had kidney transplantation during the study period were included provided they have received APD for more than 12 months. All patient demographics and baseline clinical characteristics were provided from patient registries and by the patients themselves. Body mass index (BMI) was calculated as the ratio weight/height^2^ (kg/m^2^). Systolic (SBP) and diastolic blood pressure (DBP) were measured and recorded every visit. Blood samples were collected from all patients for the biochemical and hematological data at the beginning of the study and every month and at the study termination.

### Patients’ assessment and data collection

All patients were interviewed by a cardiologist who also reviewed patient’s hospital files for demographic and disease information. The gathered information included age, sex, body weight, height, body mass index (BMI), tobacco smoking, causes of ESRD, concurrent diseases (diabetes mellitus, hypertension, ischemic and other heart diseases), APD characteristics (type, duration, dialysis adequacy), weight gain, Kt/V and residual renal function (RRF) which were recorded each visit. Erythropoietin (EPO) dosage was modified according to the patients’ need. Systolic pulmonary artery pressure (sPAP) and mean PAP (mPAP) were measured initially and on 3 months basis. ESRD cause, hemoglobin (Hb), hematocrit (Hct), serum albumin, serum creatinine (SCr), blood urea nitrogen (BUN), serum calcium (Ca), serum phosphorus (P), parathyroid hormone (PTH), C-reactive protein (CRP), total cholesterol (TC), triglyceride (TG), low-density lipoprotein cholesterol (LDL-C), high-density lipoprotein cholesterol (HDL-C), NT-proBNP and urea clearance index (KT/V) were measured monthly. In addition, serological testing was required to detect underlying connective tissue disease (CTD). Hepatitis and human immunodeficiency virus (HIV) were also performed. Keeping in mind that up to 40% of patients with idiopathic PAH have elevated antinuclear antibodies usually in a low titer (1:80). Medical assessment, determination of functional class, ECG chest x ray and ECHO cardiography were performed at baseline then every 3 months.

### Total body water (TBW) and extracellular water (ECW)

Were calculated according to the Watson’s eq. [18]:

$${\displaystyle \begin{array}{c}\textrm{TBW}\ \left(\textrm{males}\right)=2.447-0.09156\textrm{X}\ \textrm{age}+0.1074\textrm{X}\ \textrm{height}+0.3362\textrm{X}\ \textrm{weight}\\ {}\textrm{TBW}\ \left(\textrm{females}\right)=2.097+0.1069\textrm{X}\ \textrm{height}+0.2466\textrm{X}\ \textrm{weight}\end{array}}$$

Water in liters, Age in years, height in cm and weight in kg.

ECW on the other hand, was calculated using Bird’s ECV formula (ECV = weight^0.6469^ × height^0.7236^ × 0.02154) [19]. That was favored by Filler, et al. in the year 2011 [20].

### Electrocardiogram

An electrocardiogram (ECG) provided supportive evidence of PAH. We kept in mind that a normal ECG does not exclude the diagnosis. An abnormal ECG is more likely in severe rather than mild PAH. ECG abnormalities may include P pulmonale, right axis deviation, RV hypertrophy, RV strain, right bundle branch block, and QTc prolongation. While RV hypertrophy has insufficient sensitivity (55%) and specificity (70%) to be a screening tool, RV strain is more sensitive [21]. Prolongation of the QRS complex and QTc suggested severe disease [22, 23].

### Chest radiograph

In 90% of patients with IPAH the chest radiograph is abnormal at the time of diagnosis.34 Findings considered to be suggestive of PAH included central pulmonary arterial dilatation, which contrasts with ‘pruning’ (loss) of the peripheral blood vessels. Right atrium (RA) and RV enlargement in more advanced cases. A chest radiograph assisted in differential diagnosis of PAH by showing signs suggesting lung disease or pulmonary venous congestion due to left heart disease. Chest radiography helped in distinguishing between arterial and venous PAH by respectively demonstrating increased and decreased artery: vein ratios [24]. Overall, the degree of PAH in any given patient did not correlate with the extent of radiographic abnormalities. As for ECG, a normal chest radiograph did not exclude PAH.

### Echocardiographic examination

All patients were followed-up at 3-month interval for an echocardiographic examination and cardiac evaluation, and all follow-ups ended on March 31st, 2020. Echocardiographic examinations in all subjects were performed by Vivid E9 (GE Healthcare, Milwaukee, WI) by the same cardiologist. Cardiac dimensions and systolic (mild to severe) and diastolic (grades I to III) cardiac dysfunctions were assessed according to the guidelines of the American Society of Echocardiography [25]. Systolic pulmonary artery pressure was calculated as ¼ [4 (peak tricuspid regurgitant jet velocity)^2^ + right atrial pressure]. Continuous-wave Doppler echocardiography was used to estimate the sPAP when there was a tricuspid regurgitation. Mean PAP (mPAP) was estimated from sPAP by the formula; mPAP ¼ (0.61 sPAP) + 2.16 and according to the American College of Cardiology Foundation/American Heart Association 2009 expert consensus. PAH was defined in our study as systolic PAP (sPAP) > 35 mmHg or mPAP > 25 mmHg at rest [26].

### APD dialytic prescription

Our dialytic prescription consisted of Physioneal® of 1.36%, 5 l and Physioneal® 2.27, 5 l over 9-10 hours. Extraneal® 1.5 -2 l was used according to patients’ needs.

### Statistical analysis

Continuous variables are presented as mean ± SD; categorical variables were presented as frequencies and percentages. Some variables with non-normal distribution were reported as median and IQR (Tables 1 and 2), In such case a distribution free test (nonparametric test) was used. Comparisons between continuous variables were conducted by analysis of variance and *t*-test. Chi-square test was used to compare categorical variables. Related risk factors of PAH were analyzed by logistic regression analysis. *p*-Values of < 0.05 were considered as statistically significant. Data analyses were performed using Statistical Package for Social Sciences (SPSS), Version 20 for Windows (SPSS Inc., Chicago, IL, USA).

**Table 1:** Demographic, clinical and biochemical characteristics of study patients

Parameters	Values
Age (years)	52.68 + 16.33
Sex (M/F)	17/14
ESRD duration (months)	48.5 + 22.1
Duration of APD (months)	30.34 + 17.65
DM (%)	41.9
HTN (%)	67.7
Smoking (%)	29.0
History of ischemic cardiac disease (n, %)	5 (16.1)
BMI (kg/m^2^)	23.75 + 5.11
Residual urine (l/day)	0.8 + 0.3
Hemoglobin (g/dl)	8.8 + 1.4
CRP (mg/dl)	1.29 + 0.51
Albumin (g/dl)	3.21 + 0.32
Calcium (mg/dl)	8.22 + 0.95
Phosphorus	4.88 + 1.31
PTH (pg/ml)	377.6 + 174.8
TC (mg/dl)	284.9 + 46.5
TG (mg/dl)	252.3 + 33.6
LD-C (mg/dl)	169.5 + 44.8
BUN (mg/dl)	78.5 + 7.32
Kt/V, median (IQR)	1.72 (1.54-1.86)
Darbepoetin dose, median (IQR)	60 (40-80)

*ESRD* End-stage renal disease, *DM* Diabetes mellitus, *HTN* Hypertension, *BMI* Body mass index, *CRP* C-reactive protein, *PTH* Parathyroid hormone, *TC* Total cholesterol, *TG* Triglyceride, *LD-C* Low density cholesterol, *BUN* Blood urea nitrogen, *Cr* Serum creatinine

**Table 2:** Comparison of patients’ characteristics at the beginning and at the end of study

Parameters	Beginning	End	P
BUN (mg/dl)	78.5 + 7.32	38.8 + 4.9	0.004
Creatinine (mg/dl)	9.6 + 3.1	4.2 + 0.8	0.035
Dyslipidemia, n (%)	13 (41.9)	11 (35.5)	0.207
Serum Na (mEq/L), median (IQR)	131 (129-133)	134 (130-135)	0.101
Serum K (mEq/L), median (IQR)	4.8 (4.4-6.1)	3.6 (3.5-3.7)	0.041
Serum HCO3 (mEq/L), median (IQR)	16 (11-18)	23 (22-25)	0.023
PTH (pg/ml), mean + SD	377.6 + 174.8	184.3 + 55.7	< 0.001
Hemoglobin (g/dl), mean + SD	8.8 + 1.4	10.4 + 1.9	0.012
Serum albumin (g/dl), mean + SD	3.21 + 0.32	3.78 + 0.29	0.211
Volume overload	14 (45.2)	3 (9.7)	< 0.001
TBW (L)	33.81 + 7.35	28.76 + 5.48	< 0.001
ECW (L)	16.53 + 3.89	12.31 + 3.35	< 0.001
sPAP (median + SD)	40.75 + 10.61	23.55 + 9.20	< 0.001

*BUN* Blood urea nitrogen, *Na* Sodium, *K* Potassium, *HCO3* Bicarbonate, *TBW* Total body water, *ECW* Extracellular water, *sPAP* Systolic pulmonary artery pressure

## Results

The mean age of the study population (*n* = 31) was 51.23 ± 15.24 years, with 54.8% males. Mean duration on APD was 30.34 + 17.65. PAH was found in 24.2% of the patients (31 out of 128). The baseline demographic and clinical characteristics, as well as relevant laboratory tests, are presented in Table 1.

**Fig. 2:**
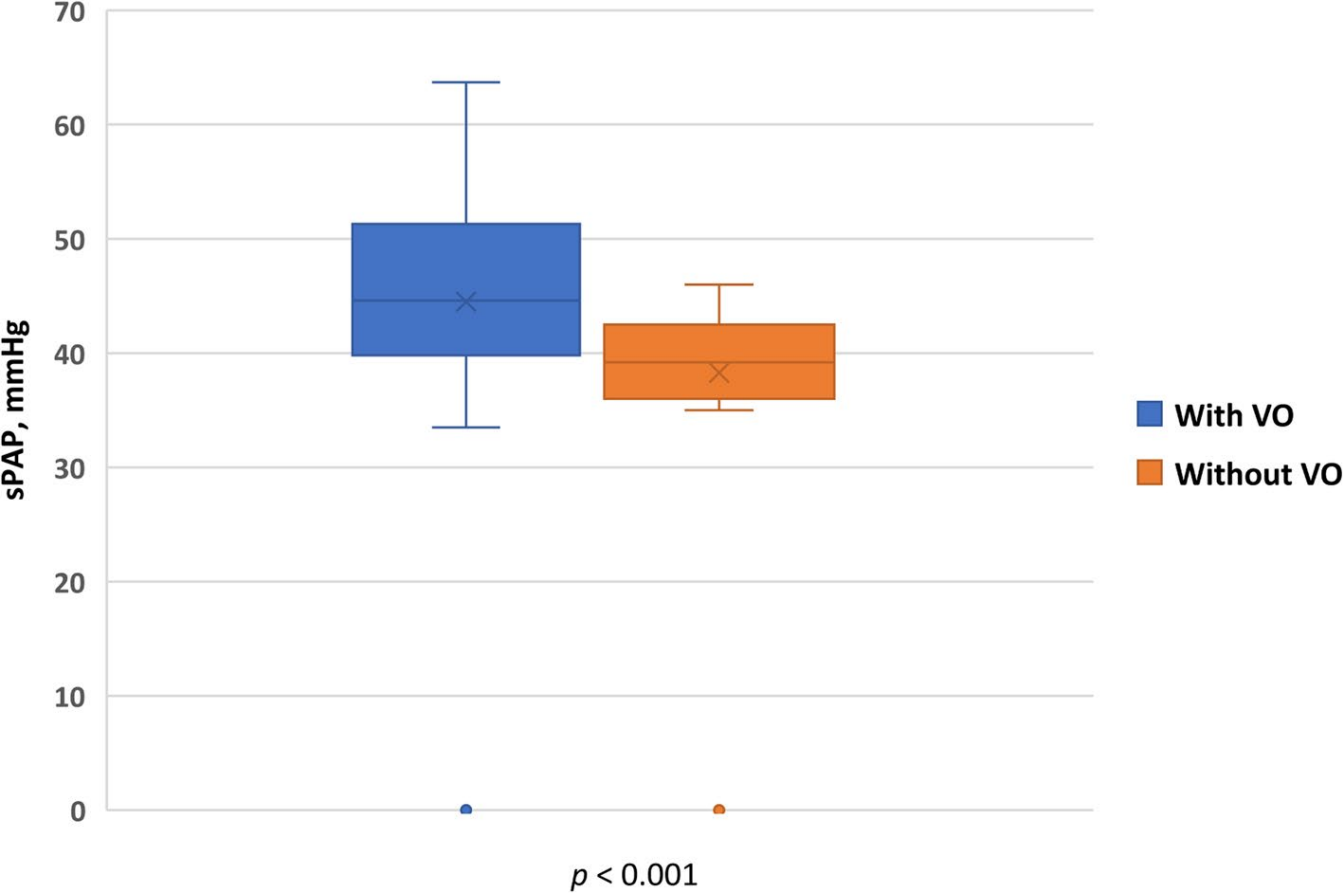
Effect of volume overload on sPAP. VO: volume overload, sPAP: systolic pulmonary arterial pressure

**Fig. 3:**
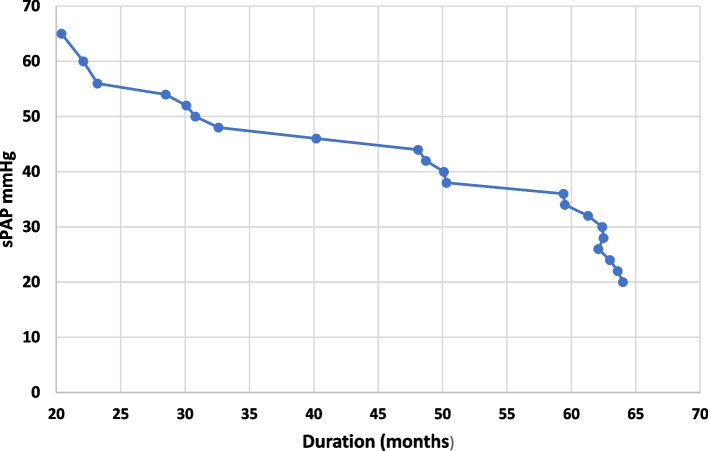
Relation between sPAP and duration on APD. APD: automated peritoneal dialysis, sPAP: systolic pulmonary artery pressure, *p* <  0.001

Mean systolic pulmonary artery pressure (sPAP) and mean pulmonary artery pressure (mPAP) were significantly higher in the APD patients at study initiation than at the end of the study (40.75 + 10.61 vs 23.55 + 9.20 and 29.66 + 11.35 vs 18.24 + 6.75 mmHg respectively, *p* = 0.001) (Tables 2 and 3). The difference in the median serum albumin levels between the two points was not statistically significant while median ejection fraction was significantly lower in patients with PAH at zero point than at study termination [31% (27-34) vs 50% (46-52)], *p* = 0.002]. Both extracellular water (ECW) and total body water (TBW), decreased significantly at the end of study (*p* <  0.001) which can reflect hydration status, and both correlated positively with the PAP (r = 0.371 and r = 0.369), *p* = 0.002). In the APD patients with PAH, no patients were hypovolemic; 14 (45.2%) of the 31 APD patients were hypervolemic and 17 (54.8%) were normovolemic. Mean systolic PAP was significantly higher in hypervolemic APD patients (39.55 + 7.21 mmHg) than in normovolemic APD patients (36.62 + 5.72 mmHg) (*p* = 0. 013). PAP correlated with left ventricular mass index (LVMI; r = 0.292, *p* = 0.001). On the other hand, it inversely correlated with hemoglobin level (r = − 0.186, *p* = 0.014), and ejection fraction (r = − 0.252, *p* = < 0.001). When performing logistic regression analysis, PAH associations were with age ≥ 65 years (OR 1.077; 95% confidence interval (CI) 1.005–1.151; *P* = 0.039); cardiovascular disease (OR 1.749; CI 95% 1.260-2.398; *P* <  0.001); diabetes (OR 1.064; CI 95% 1.026-1.104; *P* <  0.001); volume overload (Fig. 2) (OR 0.720; CI 95% 0.588-0.876; *P* <  0.001), LVMI (OR 1.380 CI 95% 1.051-1.812; *P* 0.020) and low hemoglobin levels (< 9 g/dl) (OR 1.772; CI (95%) 1.121-2.820; *P* = 0.015), but no association was found with gender (OR 0.955, CI 95% 0.842-1.080; *P* = 0.441) or serum albumin (OR 1.00; CI (95%) 0.980-1.025; *p* = 0.702). (Table 4). Duration on APD (Fig. 3) inversely correlated with PAH (r = − 267, *p* = 0.013). There were no clinically significant changes in serum sodium or chloride levels, but significant changes were noted in serum bicarbonate and potassium at the end of study (Table 2) Serum potassium < 3.5 mEq/l occurred in 4 of 31 patients (12.9%) at study termination. There was no significant difference in the residual renal function (RRF) between the start and the end of the study (0.8 + 0.3 L vs. 0.77 + 0.4 L, *p* = 0.461). Ultrafiltration (UF) and Kt/V: the medium (IQR) UF in our patients was 1100 ml (840-1440 ml) per session. The median (IQR) Kt/V was 1.72 (1.67-1.88).

**Table 3:** Comparison of echocardiographic findings between the beginning and end of the study

Parameters	Initial findings	End of study	*p*
LVEF (%) [median (IQR)]	31 (27-34)	50 (46-52)	0.002
Systolic dysfunction			
Mild, n (%)	8 (25.8)	20 (64.5)	< 0.001
Moderate, n (%)	16 (51.6)	9 (29.0)	< 0.01
Severe, n (%)	7 (22.6)	2 (6.5)	< 0.01
Diastolic dysfunction			
Grade I, n (%)	10 (32.3)	19 (61.3)	< 0.01
Grade II, n (%)	21 (67.7)	12 (38.7)	< 0.01
Grade III, IV, n (%)	0 (0)	0 (0)	–
Right atrial dilatation, n (%)	24 (77.4)	11 (35.5)	< 0.001
Right ventricular dilatation, n (%)	26 (83.9)	12 (38.7)	< 0.001
Left atrial dilatation, n (%)	16 (51.6)	7 (22.6)	< 0.001
Increased left ventricular wall thickness, n (%)	11 (33.3)	9 (29.0)	0.183
Septal thickness, cm (mean + SD)	2.2 + 0.3	1.4 + 0.2	< 0.001
PE, n (%)	9 (29.0)	4 (12.9)	0.035
sPAP (median + SD)	40.75 + 10.61	23.55 + 9.20	< 0.001
mPAP (median + SD)	29.66 + 11.35	18.24 + 6.75	< 0.001

*LVEF* Left ventricular ejection fraction, *IQR* Interquartile ratio, *PE* Pericardial effusion, *sPAP* Systolic pulmonary artery pressure, *mPAP* Mean pulmonary artery pressure

**Table 4:** Logistic regression analysis for factors involved in PAH in APD patients

Variables	OR	95% CI	*p*
Age > 65 years	1.077	1.005-1.151	0.039
Volume overload	0.720	0.588-0.876	< 0.001
Female gender	0.955	0.842-1.080	0.441
Hemoglobin	1.772	1.121-2.820	0.015
Serum albumin	1.00	0.980-1.025	0.702
Diabetes mellitus	1.064	1.026-1.104	< 0.001
Cardiac disease	1.749	1.260-2.398	< 0.001
LVMI	1.380	1.051-1.812	0.020

*OR* Odd ratio, *CI* Confidence interval, *LVMI* Left ventricular mass index

ECHO findings are presented in Table 3. There were favorable and significant changes in systolic and diastolic functions at the end of study (*p* <  0.001 and *p* <  0.01). Significant improvement was also noted in right atrial, right ventricular and left atrial measurements (*p* <  0.001). In addition, significant changes were noted in the septal thickness (*p* <  0.001) but not in the left ventricular posterior wall thickness (*p* = 0.183) at the end of study compared with the initial values (Table 3). Specific ECG changes were recorded in 5 (16.1%) and significant chest x-ray findings were observed in 10 (32.3%) patients. At the end of the study (5 years), 2 patients out of 31 (6.5%) died; the cause of death in both was complications of acute myocardial infarction.

## Discussion

Pulmonary artery hypertension (PAH) is a complex syndrome defined by an elevated mean pulmonary artery pressure on right heart catheterization (RHC) [27, 28]. Over the last 15 years, it has been increasingly recognized that chronic kidney disease (CKD), especially end-stage renal disease (ESRD), is a risk factor for multifactorial pulmonary hypertension [28–30]. The mechanism is poorly understood but is likely a combination of chronic volume overload with pulmonary vascular remodeling, diastolic dysfunction, elevated cardiac output due to an arterio-venous fistula (AVF) or chronic anemia, and chronic inflammation (11). Furthermore, the presence of PAH in ESRD has been associated with worse clinical outcomes for patients [31]. Many researchers have studied PAH in ESRD on hemodialysis, but few studies (only 11) have investigated the same with PD as stated in a recent metanalysis [32]. The sPAP used to diagnose PAH varied among studies; but ranged from > 30 mmHg to > 45 mmHg [32]. Compared with hemodialysis (HD), the prevalence of PAH was much less in ESRD patients receiving treatment with PD. The median prevalence of PAH was 38% (range 8 to 70%) among patients undergoing any type of dialysis, 40% (range 16–70%) among patients undergoing HD, and 19% (range 8–37%) among patients undergoing PD [32]. In our study the prevalence of PAH (24.2%) was in line with those reported in previous studies [33–35]. Studies from the Middle East and North Africa [33–37] had a pooled prevalence among patients undergoing any type of dialysis of 38% (95% CI 30–45%), among patients receiving HD of 42% (95% CI 35–50%), and among patients receiving PD of 15% (95% CI 9–21%). Studies from East Asia had a pooled prevalence among patients undergoing any type of dialysis of 35% (95% CI 27–44%), among patients receiving HD of 44% (95% CI 38–51%), and among patients receiving PD of 24% (95% CI 14–34%) [16, 17, 33–35, 38–46]. Pathogenesis of PAH in ESRD has not been completely elucidated and the mechanisms leading to the disease are still under investigation [39, 40]. A cross-sectional study by Unal et al. demonstrated a close association between hypervolemia and PAH by using bioimpedance analysis [17]. Similarly, Agarwal et al. speculated that pulmonary hypertension may occur in response to chronic volume overload [13]. Interestingly, the study showed that fluid overload was significantly higher in dialysis patients with PAH than those without PAH. Also, sPAP and TBW-ECW levels and the frequency of PAH were significantly reduced after dialysis, and a significant positive correlation was found between sPAP and volume overload. It is possible that chronic fluid overload associated with hyperdynamic circulation causes elevated right atrial pressure, elevated mean pulmonary artery pressure as a consequence of increased pulmonary blood flow. In our study, volume overload was a definite risk factors for PAH and ECHO abnormalities as demonstrated by univariate and multivariant analyses. Other factors that can contribute to the development of PAH by increasing cardiac output are anemia and low albumin, [17, 28, 42, 47–51]. Our analysis, however, did not confirm a relation between serum albumin levels and PAH and this could probably be due to the small sample size and the fact that there were no significant differences in albumin levels between the beginning and end of the study. Contrary to previous reports [13, 17, 28, 42–47] duration of dialysis inversely correlated with the risk of PAH and this was proved by both correlation coefficient (r = − 267, *p* = 0.013) and by multivariant analysis (*p* = < 0.001), which may not be attributed only to one variable, but getting other factors in light. Reviewing literature showed significant relationship between impaired production and decreased responsiveness of nitric oxide in pulmonary endothelial vascular smooth muscle in patient with high PAP [47]. Endothelin-1 is a potent vasoconstrictor that had an important role in development of PAH [48], increase in endothelial activities has been reported in chronic renal failure [49]. Rubin et al., reported a significant drop of PAP in 19-year-old hemodialysis patient after treatment with Bosentan (an endothelial receptor antagonist) [50]. The cytokines in particular (tumor necrosis factors alpha, endothelial-1, atrial natriuretic peptide (ANP), brain natriuretic peptide (BNP) and interleukin-1) have been shown to induce pulmonary angiogenesis, fibroblast proliferation and apoptosis of cardiac myocytes [51, 52]. High level of BNP has been reported by as poor prognostic factor in patients with PAH [52–56]. There is high body of evidence indicating that PD has the capability to remove small and middle-size molecules [30]. The molecular weight of TNF alpha is about 17KDa and that of other myocardial depressant factors ranges between 700 and 800 [55]. Thus, the removal of these small and middle weight cytokines by PD is probably another important factor in prevention of PAH in PD patients. In addition, PD is more or less considered as normal physiological process with no hemodynamic disturbances and no A-V access that can augment PAH in dialysis patients which could explain the low prevalence of PAH in PD patients and the improvement of PAP with time in PD patients [30]. Risk factors for PAH in our study were found to be age (> 65), volume overload, cardiovascular disease and low hemoglobin levels (defined as 8 g/dl or less). In the younger than 65 years of age, when adjusting for age, PAH significantly improved at the end of the study. Although RRF in our study did not vary significantly between the beginning and end, we do believe that RRF plays a major role in APD adequacy and outcomes. RRF might have a significant impact on the initial as well as the end volume status in our report. Fluid overload is a common and serious problem that leads to severe complications in HD patients and has a great impact on the pathogenesis of cardiovascular disease and PAH. (76). We think that volume control in our APD patients played a role in reducing PAP. In our report, old age (> 65) was suggested as a possible risk factor for PAH and demonstrated by analysis. PAH is increasingly recognized in the elderly population; however, its causes and characteristics in those population are not well established [57–61]. A report from a multinational European registry found 63% of patients in a cohort of IPAH were aged ≥65 years [60–62] and an analysis of incident cases of PAH in the United Kingdom and Ireland reported 13.5% of patients were diagnosed with PAH at age ≥ 70 years [61] and PAH associated with heart disease and vascular calcifications even with preserved ejection fraction is an increasingly recognized cause of PAH in older adults [62–64]. Whatever mechanisms causing PAH in elderly, we suggest that PD is a reasonable and effective option for ESRD elderly patients based on the results in our study. Since PAH is associated with significant morbidity and mortality in ESRD patients, its prevention and early diagnosis and treatment is of great importance. In patients who are at known risk for development of PAH, such as those with pre-existing moderate to severe systolic/diastolic cardiac dysfunction, changing the dialysis type from HD to PD may be a reasonable option to prevent PAH or to prevent further elevation of PAP. The limitations of this study are the small size of study population, and the fact that the peritoneal membrane transport characteristics of the patients were not evaluated. However, the fact that it is a long-term study of incident patients with a minimum time of 1 years and up to 5 years follow-up, the therapy being provided by a single dialysis supplier, and the thorough quality control used for collecting data and handling the database may authenticate our report.

## Conclusion

Automated PD seems to be a reasonable, effective and safe option for treating patients with ESRD and PAH. It may also be effective in improving LVEF and cardiac functions. Long term outcome is favorable, and mortality is low with this modality. Further studies with a larger cohort are encouraged.

## Data Availability

All data and materials are presented in detail within the manuscript.

## Abbreviations

APD: Automated peritoneal dialysis
AVF: Arteriovenous fistula
BMI: Body mass index
BNP: Brain natriuretic peptide
BUN: Blood urea nitrogen
Ca: Serum calcium
CAPD: Continuous ambulatory peritoneal dialysis
CI: Confidence interval
CKD: Chronic kidney disease
CO: Cardiac output
CRP: C-reactive protein
CTD: Connective tissue disease
DBP: Diastolic blood pressure
ECG: Electrocardiogram
ECW: Extracellular water
EPO: Erythropoietin
ESRD: End-stage renal disease
HD: Hemodialysis
HDL-C: High density lipoprotein cholesterol
HIV: Human immunodeficiency virus
HR: Hazard ratio
IPAH: Idiopathic pulmonary artery hypertension
IQR: Interquartile ratio
Kt/V: Urea clearance index
LDL-C: Low density lipoprotein cholesterol
LVEF: Left ventricular ejection fraction
LVMI: Left ventricular mass index
mPAP: Mean pulmonary artery pressure
NT ProBNP: N-terminal pro-brain natriuretic peptide
P: Serum phosphorus
PAH: Pulmonary artery hypertension
PD: Peritoneal dialysis
PTH: Parathyroid hormone
r: Correlation coefficient
RRF: Residual renal function
RV: Right ventricle
SBP: Systolic blood pressure
SCr: Serum creatinine
SD: Standard deviation
sPAP: Systolic pulmonary artery pressure
TBW: Total body water
TC: Total cholesterol
TNF: Tumor necrosis factor
VO: Volume overload
